# Adequacy of antenatal care and its relationship with low birth weight in Botucatu, São Paulo, Brazil: a case-control study

**DOI:** 10.1186/1471-2393-14-255

**Published:** 2014-08-01

**Authors:** Cátia Regina Branco da Fonseca, Maria Wany Louzada Strufaldi, Lídia Raquel de Carvalho, Rosana Fiorini Puccini

**Affiliations:** Department of Pediatrics, Botucatu Medical School, Paulista State University, UNESP, Botucatu, São Paulo Brazil; Department of Pediatrics, Federal University of São Paulo, UNIFESP, São Paulo, São Paulo Brazil; Department of Biostatistics, Institute of Biosciences, Paulista State University, UNESP, Botucatu, São Paulo Brazil

**Keywords:** Low birth weight, Antenatal care, Prenatal care, Adequacy of health care, Health evaluation, Public health system

## Abstract

**Background:**

Birth weight reflects gestational conditions and development during the fetal period. Low birth weight (LBW) may be associated with antenatal care (ANC) adequacy and quality. The purpose of this study was to analyze ANC adequacy and its relationship with LBW in the Unified Health System in Brazil.

**Methods:**

A case-control study was conducted in Botucatu, São Paulo, Brazil, 2004 to 2008. Data were collected from secondary sources (the Live Birth Certificate), and primary sources (the official medical records of pregnant women). The study population consisted of two groups, each with 860 newborns. The case group comprised newborns weighing less than 2,500 grams, while the control group comprised live newborns weighing greater than or equal to 2,500 grams. Adequacy of ANC was evaluated according to three measurements: 1. Adequacy of the number of ANC visits adjusted to gestational age; 2. Modified Kessner Index; and 3. Adequacy of ANC laboratory studies and exams summary measure according to parameters defined by the Ministry of Health in the Program for Prenatal and Birth Care Humanization.

**Results:**

Analyses revealed that LBW was associated with the number of ANC visits adjusted to gestational age (OR = 1.78, 95% CI 1.32-2.34) and the ANC laboratory studies and exams summary measure (OR = 4.13, 95% CI 1.36-12.51). According to the modified Kessner Index, 64.4% of antenatal visits in the LBW group were adequate, with no differences between groups.

**Conclusions:**

Our data corroborate the association between inadequate number of ANC visits, laboratory studies and exams, and increased risk of LBW newborns. No association was found between the modified Kessner Index as a measure of adequacy of ANC and LBW. This finding reveals the low indices of coverage for basic actions already well regulated in the Health System in Brazil. Despite the association found in the study, we cannot conclude that LBW would be prevented only by an adequate ANC, as LBW is associated with factors of complex and multifactorial etiology. The results could be used to plan monitoring measures and evaluate programs of health care assistance during pregnancy, at delivery and to newborns, focusing on reduced LBW rates.

## Background

Birth weight reflects gestational conditions and development in the fetal period. Low birth weight (LBW), defined by the World Health Organization (WHO) as birth weight less than 2,500 grams [1], may be a result of premature birth and/or intrauterine growth restriction. Research and health programs have focused on LBW newborns, as they are particularly vulnerable to the impacts of environmental and social conditions. LBW has a complex etiology, and LBW newborns experience higher mortality rates. Therefore, studies on factors affecting LBW are of great interest to the Brazilian Health System [2, 3] and to many middle- and low-income countries in the world [4]. Much research has investigated LBW, with particular attention paid to its etiology and population disparities, consequences, and means of prevention [5–7].

Over the last few years, antenatal and childbirth care in Brazil has improved, access to health services has been extended [8], and social inequality has been reduced [9]. However, the Brazilian official data from the last few years show that LBW rates have risen and are higher in more developed regions. In Brazil, the rate of LBW (per 1,000 live births) was 7.7 in 2000. It ranged from 8.24 in 2004 to 8.5 in 2008. In an effort to understand this unexpected finding, a number of studies were conducted and no significant reduction in LBW prevalence was found among a number of Brazilian cities [10]. Regional differences have been attributed to the availability of prenatal and perinatal care rather than social conditions [10]. Understanding these differences, as well as LBW determinants in Brazil, is crucial for planning actions to enhance the Public Health System, and by extension reducing the rate of LBW and improving health outcomes for LBW infants [11].

To improve care and build on actions already established, the Program for Prenatal and Birth Care Humanization (PPBH) was implemented in 2000 along with additional strategic actions to improve the quality of care of the pregnant woman and fetus [12]. All the health team in the Basic Health Unit (including physicians and nurses) should be qualified to perform the daily activities of this Program. Monitoring of this activities is performed by using a System of National Information on Prenatal (SISPRENATAL, in Portuguese language acronym), with an increased transfer of financial resources for the city which meets the goal of prenatal care service, that is, since the registration of the pregnant women until the consultation during puerperium following the minimum recommended guidelines [12].

The PPBH guidelines recommend the following: ANC by 17 weeks gestation; six physician visits minimum (one, two and three visits in the first, second and third trimester, respectively); measure the symphysis-fundal height; perform a number of laboratory tests during the first trimester (blood typing; serology testing for toxoplasmosis, Human Immunodeficiency Virus (HIV), syphilis and hepatitis B; stool culture test; complete blood count; urine culture, and; fasting blood glycemia); oncotic colpocytology; tetanus vaccination, and; establishment of groups of pregnant women to be assisted on ANC, breastfeeding and newborn care [12]. The obstetric ultrasound is not considered as being a routine exam in PPBH, and according to the Ministry of Health, it is not necessary in normal prenatal care, except for high-risk pregnant women [13]. However, this exam was included in the Municipal Program of Prenatal Care in the Basic Units of the study city, and at least one exam was recommended at the beginning of gestation and in the third trimester, if necessary – obstetric referral focusing the investigation on fetal abnormalities [14].

Moreover, the program raised the discussion about antenatal practices and their conceptual basis [15]. Major actions sought to establish a national model that would guide the care provided, therefore ensuring pregnant women the right of trusted and qualified care during gestation, childbirth and the postpartum period [7, 12, 16].

The correlation between inadequate ANC and increased rate of maternal and perinatal morbidity has been known since as early as 1914, when studies reported that timely detection and prompt treatment of pregnancy complications considerably reduced perinatal mortality from a variety of causes, including prematurity, small for gestational age (SGA) and LBW newborn [17–19].

Some studies suggest an association between adequacy of ANC and birth outcomes and several prenatal care indices are used to evaluate this association. Each of these indices makes use of information on the time of prenatal care initiation, the total number of prenatal visits, and gestational age at birth. Therefore, prenatal care is categorized according to different analyses [20]. The most widely-used indices include the Kessner Index [21], the Graduated Prenatal Care Utilization Index (GINDEX) [22] and the Adequacy of Prenatal Care Utilization (APNCU) [23], and have been routinely used in the analysis of birth outcomes. According to Heaman et al. (2008), the association between inadequate ANC and preterm birth weight and LBW has varied depending on the selection of the prenatal care utilization index, and therefore careful consideration of the methodological underpinnings and limitations of prenatal indices is required [24].

In the last 15 years in Brazil, an increase in ANC coverage and number of visits per pregnant woman has been observed, and as of 2009, the percentage of pregnant women with no access to ANC was less than 2% [25]. However, the prevalence of LBW in Brazil has been stable since 2000 onward [26]. A reduction in the frequency of intrauterine growth restriction has been reported, and this finding may have offset the negative effect of the increased frequency of preterm deliveries on birth weight [26].

The preterm birth rate in Brazil was 4.8 live births per 100 births in 1998, and 5.9 live births per 100 births in 2001 [25]. The preterm birth rate increased from 5.4% to 7.3% and 3.4% to 7.4% in the Southern and Southeastern regions between 1994 and 2005, respectively [27]. Studies conducted in the Northern region between 1984 and 1998, revealed that the prevalence of preterm births increased from 3.8% to 10.2% [28]. There are few data on rates of SGA newborns in Brazil. Zambonato et al. (2004) reported values of 13.1% for this parameter in a study conducted in a southern city of Brazil in 1996 [29].

The extent to which medical interventions such as cesarean sections have contributed to the increase in preterm infants has been much debated in Brazil. Some studies report an association between these parameters, while others report that preterm births have increased equally for both vaginal and cesarean deliveries [28]. Moreover, the Brazilian woman’s preference for non-medically indicated cesarean sections is associated with a higher socioeconomic level, white ethnicity, higher education and higher adequacy of ANC [30]. Regardless of socioeconomic level, the demand for cesarean section seems to be based on the belief that quality of obstetric care is closely associated to the technology used in the surgical birth [27], however, it may be responsible for the increased preterm an LBW newborn rates in more developed regions in Brazil, such as the South and Southeast [27, 30].

Brazil is a country of great size and widespread regional and social inequalities. The Unified Health System (SUS, the Portuguese-language acronym) was implemented by the 1988 Constitution, and is based on the principle of health as a citizen’s right and duty of the state. The development of primary health care, or basic care as it is called in the SUS, has been the subject of much attention in Brazil. The primary health care model aims to provide universal access and comprehensive health care, coordinating and expanding coverage to more specialized levels of care (e.g. specialized care and hospital care), and implementing intersectoral actions for health promotion and disease prevention [26].

The Brazilian Health system has implemented several information systems addressing different areas: epidemiology, demographics, service production and other functionalities. To record the Brazilian experience as accurately as possible, the SUS has developed a number of important national programs in the health area in the last 30 years [31]. They are information systems with free public access through passwords for health professionals, and are detailed below:

- The System of Information on Live Births (SINASC, the Portuguese language acronym) - registers the demographic and health experience in the country, providing information on live births;
- The Department of Informatics in the SUS (DATASUS, the Portuguese language acronym) - registers, compiles and disseminates data on health from the SUS;
- The State System Foundation for Data Analysis (SEADE, the Portuguese language acronym) -. Because São Paulo is the biggest state in Brazil, this system of information is able to present itemized data of cities, registering a wide and updated range of data and indicators that are indispensable for comprehension of this rich, diversified and complex region of the country.

The purpose of this study was to analyze ANC adequacy and its relationship with LBW in the SUS of Brazil.

## Methods

We conducted a case-control study in Botucatu, São Paulo, Brazil, using data from the Live Birth Certificate (LBC), a secondary data source within the SINASC (public and free system available in Brazil), and the official medical records of pregnant women, a primary data source, recorded from the beginning of 2004 to the end of 2008. To access patient medical records in the Public University Hospital and Basic Health Units, permissions were given by the responsible for the sectors of Neonatology and Obstetrics of the Clinics Hospital and by the responsible for the Municipal Department of Health in Botucatu city. All permissions were provided as official documents for the Research Ethics Committee at the time of the approval of the Project.

ANC was performed in 16 Basic Health Units (BHU), which are primary health care centers, and outpatient clinics of the Public University Hospital – Clinics Hospital (CH), which are secondary health care centers. The follow-up of high-risk pregnancies was performed in the outpatient clinics.

Births took place in two maternity hospitals in the city, the CH and a hospital affiliated with the SUS.

According to data from governmental sources (DATASUS, SINASC and SEADE), a total of 8,442 live newborns including 860 LBW newborns were born during the study period [25, 32]. All LBW newborns were included in this study.

**Figure 1:**
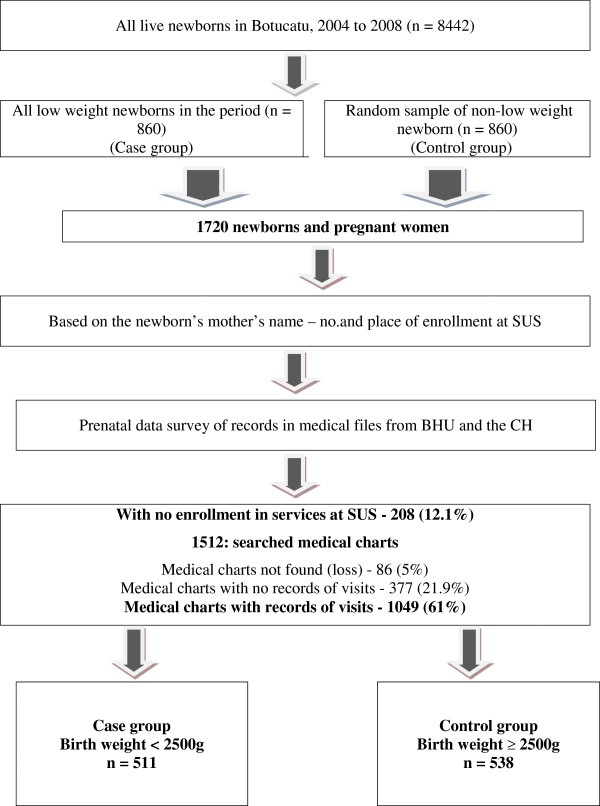
**Flowchart of the study population, data source and study groups.** Source: Information System of Live Births and Records of pregnant women in Primary Health Care Units and Clinics Hospital. A total of 8,442 live newborns including 860 LBW newborns were born during the study period. The case group consisted of all LBW newborns, as defined by the WHO [1], that were born during the study period. The control group consisted of a random sample of 860 live newborns weighing greater than or equal to 2,500 grams. After establishing the two groups, the analysis of ANC adequacy was performed for all newborns that were recorded on the medical charts of pregnant women that received ANC in health units registered in the SUS.

The case group consisted of all LBW newborns, as defined by the WHO [1] less than 2,500 grams, that were born during the study period. The control group consisted of a random sample of 860 live newborns weighing greater than or equal to 2,500 grams. For each year of the study, the same number of newborns was referred to the case group and the control group. The control group was assembled by randomly sampling from a list of LBC numbers. The number of the medical chart of each pregnant woman was located through the Municipal Information System of Health, and later matched to her medical chart in the BHU and CH (Figure 1). There was no matching of controls to cases based on maternal characteristics, as one of the study aims was identify maternal risk factors for LBW.

**Figure 2:**
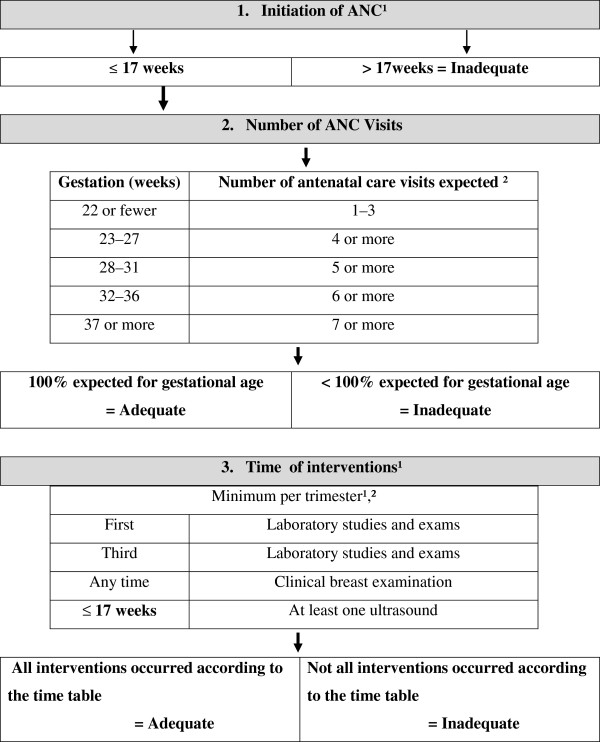
**Outline of adequacy of ANC and content of care.** For analyses of adequacy and content of ANC, three measurements were considered. 1. Adequacy of the number of ANC visits adjusted to gestational age– seven visits or more when gestational age was equal to or more than 37 weeks was deemed adequate. For gestational age less than 37 weeks, the number of expected ANC visits was adjusted by gestational age; 2. The modified Kessner Index– defined according to the number of ANC visits and the initiation of ANC, according to the Brazilian proposal in the PPBH [12, 23, 33]. ANC was deemed adequate when 100% of expected visits were attended and ANC began by 17 weeks gestation, while ANC was deemed inadequate when less than 100% of expected visits were attended or ANC began after 17 weeks gestation; 3. The ANC laboratory studies and exams summary measure. Adequacy based on PPBH and Municipal Protocol [12, 14]. ANC was considered adequate when all three criteria were met, and inadequate when any one of the three criteria was not met.

After establishing the two groups, the analysis of ANC adequacy was performed for all newborns that were recorded on the medical charts of pregnant women that received ANC in health units registered in the SUS (Figure 1). The focus of this study was the evaluation of SUS users. For analyses of adequacy and content of ANC, three measurements were considered. An outline is shown in Figure 2.

1. *Adequacy of the number of ANC visits adjusted to gestational age –* seven visits or more when gestational age was equal to or more than 37 weeks was deemed adequate [12]. For gestational age less than 37 weeks, the number of expected ANC visits was graded by gestational age.
2. *The modified Kessner Index* – defined according to the number of ANC visits and the initiation of ANC. The modified Kessner Index was used for the Brazilian proposal in the PPBH [12, 23, 33]. According to the modified Kessner Index, ANC was deemed adequate when 100% of expected visits were attended and ANC began by 17 weeks gestation, while ANC was deemed inadequate when less than 100% of expected visits were attended or ANC began after 17 weeks gestation.
3. *The ANC laboratory studies and exams summary measure* – defined according to: a) the procedures routinely recommended in Brazil [12] such as complementary exams in the first and third trimesters of gestation (Figure 2; Table 1); b) clinical breast examination recorded in any medical visit during the gestational period; and c) at least one ultrasound at the beginning of follow-up until the 17th week [14] or at any time in high risk gestation [13]. ANC was considered adequate when all three criteria were met, and inadequate when any one of the three criteria was not met.

**Table 1:** **Complementary exams routinely recommended in Brazil according to trimesters of gestation**

Trimester	Exams
First	Cervical colpocytology and laboratorial exams: blood typing; serology for Human Immunodeficiency Virus (HIV), Syphilis and Hepatitis B; stool culture test; complete blood count; urine culture and fasting blood glycemia
Third	Serology for Syphilis and AIDS, and if necessary for Toxoplasmosis and Hepatitis B; urine culture and fasting blood glycemia
First, Second or Third	Ultrasound – at least one

Source: Program for Prenatal and Birth Care Humanization and Municipal Protocol [12, 14].

All data included in the indices above were collected from the medical records of pregnant women, and, as laboratory studies and exams are mandatory in the first and third trimesters (Table 1) according to PPBH recommendations, a Yes/No rating system was used to evaluate the performance of the exams [34, 35].

### Statistical analysis

A database was built using Microsoft Office Excel 2007 (Washington, USA), and analyzed by the Statistical Analysis System (SAS, North Carolina, USA), version 9.2.

The chi-square test was used to evaluate the association among variables. The significance level was 5% (alpha = 0.05) for rejection of the null hypothesis.

In the multivariable analysis, a logistic regression model was used to investigate LBW as a dependent variable in relation to other variables of interest. For multivariable analyses, p-value, calculated by Wald test, was considered significant for p ≤ 0.05. To determine the effect of each variable on low birth weight, odds ratios with 95% confidence intervals were calculated using the logistic regression model with *k* explanatory variables [36]. The minimum power of the statistical test was 80%.

Following the ethical principles established in the Helsinki Declaration, this study was evaluated and approved by the Research Ethics Committee (REC) of Botucatu Medical School/UNESP (3372/2009) and the REC of the Federal University of São Paulo/UNIFESP (0280/2010).

The manuscript is in conformity with the Strengthening the Reporting of Observational studies in Epidemiology (STROBE guidelines), and all recommendations were included in the study.

## Results

A total of 1720 protocols for all newborns were filled out, for which 1049 charts containing records of pregnant women with ANC registered with the SUS were analyzed (Figure 1). Under the guidance of PPBH [12] for ANC, if a procedure had not been recorded, it was assumed that it had not been performed.

The study groups consisted of 511 and 538 newborns in the case and control groups, respectively (Table 2). No statistically significant difference was found between the number of newborns per year by study groups (p = 0.49).

**Table 2:** **Distribution of the number of newborns per year according to the study groups**

GROUP	Number of newborns per year					Total	
	2004	2005	2006	2007	2008		***p***
I – Case (<2500 g)	112	96	98	107	98	511	0.49
II – Control (≥2500 g)	108	97	122	122	89	538	
Total	220	193	220	229	187	1049	

Source: SEADE – database from São Paulo state [32].

Forty-seven medical records lacked information on the trimester of ANC initiation, 31 (6.1%) in the case group and 16 (2.9%) in the control group (Table 3). No information on some of the laboratory studies and exams was found for 223 (43.6%) of pregnant women in the case group, and for 253 (47.0%) of the pregnant women in the control group (Table 3). There was no statistically significant difference in the size of the two groups due this loss of information (p = 0.49).

**Table 3:** **Content and procedures of antenatal care by group**

ANC procedures	Case group				Control group				***p***
	(<2500 g)				(≥2500 g)				
	Mean		SD	Range	Mean	SD	Range		
Number of ANC visits	16.9		6.9	2–41	18	6.9	4 - 44		**0.001**
Number of gestational weeks at the first ANC visit^1^	13.5		6.2	4 - 36.4	13.6	8.2	1.6-33.6		0.810
**Number of ANC visits**	**N = 511**		%		**N=538**		%		***p***
Adequate	351		68.7		433		80.5		**< 0.001**
Initiation of ANC¹	**≤ 17 weeks**		**> 17weeks**		**≤ 17 weeks**		**≤ 17 weeks**		***p***
	N	%	N	%	N	%	N	%	0.85
	370	77.1	110	22.9	399	76.4	123	23.6	
**Laboratory studies and exams**	**N= 511²**		**%**		**N= 538²**		**%**		***p***
Adequate	272		94.4		281		98.6		**0.007**

Source: Records of pregnant women in Primary Health Care Units and Clinics Hospital.

^1^No information Case group 31 (6.1%); Control group 16 (2.9%).

^2^No Information Case group 223 (43.6%); Control group 253 (47.0%).

Mean, standard deviation (SD) and range of the number of ANC visits and gestational age at the beginning of ANC are shown in Table 3. A statistically significant difference was found between groups for the number of ANC visits attended.

**Figure 3:**
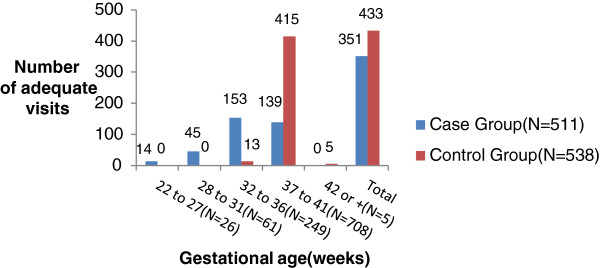
**Distribution of frequency of visits according to gestational age and adequacy** ^**1**^ **in groups.** Source: Records of pregnant women in Primary Health Care Units and Clinics Hospital. Proposition of adequacy adapted for PPBH guidelines [12], according the gestational age in weeks, in the case and control groups.

Concerning adequacy of the number of ANC visits by gestational age (Figure 3), a statistically significant difference was found between the case and control groups (p < 0.001), with a lower percentage of adequacy in the group with LBW newborns, OR = 1.78, 95% CI 1.32-2.34. This finding shows that the number of inadequate ANC visits is associated with LBW, and therefore a higher probability of LBW newborns.

The modified Kessner Index showed lower percentage of adequacy of ANC associated with LBW, 64.4% and 61.3% for case and control groups, respectively (*p*-value = 0.21), the statistical analysis shows that this measurement is not associated with increased risk of LBW (Table 4). Analyzing the content of the modified Kessner Index (Table 3), the difference found between the groups concerning number of adequate visits adjusted to gestational age was statistically significant. However, no difference was found concerning beginning of antenatal follow-up between the groups, although a higher percentage of adequacy was found for the LBW group.

**Figure 4:**
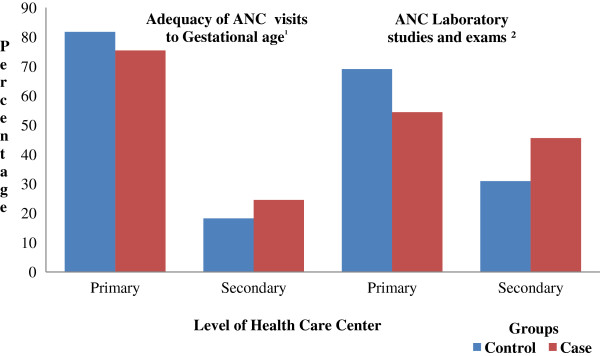
**Distribution of primary and secondary health care level of ANC and adequacy measures in groups.** Source: Records of pregnant women in Primary Health Care Units and Clinics Hospital. ^1^p value = 0.03; ^2^p-value < 0.01. Analysis of ANC in the groups according to primary or secondary health care level – BHU and outpatient clinics in CH, respectively, and the first and third measures of quality of ANC proposed in this study.

In the analysis of ANC according to primary or secondary health care level – BHU and outpatient clinics in CH, respectively) shown in Figure 4, a statistically significant difference was found between groups according to levels of care and the first and third measures of quality of ANC. The highest adequacy of ANC was found in the primary health care. For the first measure, adequacy was 75.4% and 81.8% in the Case and Control groups (p-value = 0.03), respectively. For the third measure, adequacy of ANC was 54.4% and 69.0% in the Case and Control groups (p-value < 0.01), respectively. A higher adequacy of first and third measures of quality of ANC for the Control group in primary health care level, and for the Case group in secondary health care level was statistically significant (p-value <0.05) (Figure 4).

**Table 4:** **Adequacy of antenatal care by group and criteria**

Criterion of adequacy	Group				p
	Case group		Control group		
	(<2500 g)		(≥2500 g)		
	Adequate		Adequate		
	N = 511	%	N = 538	%	
1. Number of visits^1^	351	68.7	433	80.5	**< 0.001**
2. Modified Kessner Index^2^	299	64.4	330	61.3	0.210
3. Antenatal care^3^	272	94.4	281	98.6	**0.007**

**Source:** Records of pregnant women in Primary Health Care Units and Clinics Hospital.

^1^Adequacy based on PPBH [12]; ^2^No information Case group 31 (6.1%); Control group 16 (2.9%);

^3^No Information Case group 223 (43.6%); Control group 253 (47.0%).

According to the ANC laboratory studies and exams summary measure, which includes laboratory studies and exams, a lower percentage of adequacy was found for the case group. Table 3 shows the set of criteria used for evaluation of adequacy and content of ANC. According to our analyses, this measurement was associated with increased risk of LBW newborns (OR = 4.13, 95% CI 1.36-12.51).

## Discussion

In this study, two out of three measurements used to determine the adequacy of ANC were shown to be associated with LBW, as follows: Adequacy of the number of ANC visits adjusted to gestational age and the ANC laboratory studies and exams summary measure. This finding corroborates the hypothesis that an inadequate number of antenatal visits is associated with LBW. Observational studies have demonstrated the benefits of this assistance, and associations have been observed between a higher number of visits and more favorable outcomes, for example, adequate weight of newborns [37, 38]. Moreover, socioeconomic inequalities, demographic factors and behavioral risk factors are still important factors associated with inadequate ANC in developing countries [39].

The relationship between LBW and the procedures included in ANC still requires investigation. According to Silveira and Santos (2004), study design, the chosen indicator of antenatal adequacy, and data sources and analyses can impact study results and lead to different conclusions on the same issue, making study comparison difficult [40]. In this study, a re-analysis was performed so that eliminating the categorical element, appropriate number of ANC visits by gestational age, a component of the third criterion of ANC quality, the result was changed from no association to significant association of laboratory studies and examination with LBW.

The criterion used for evaluation of adequacy and content of ANC is crucial, and the more specific it is, the better it reflects the local reality of the study and provides information for discussion on the care delivered [34, 41]. In this study, the modified Kessner index was used because it is one of the main indices currently reported in the literature to measure adequacy of ANC [21–23]. Different definitions and limitations of the main indices have been reported that are separate from the method used to evaluate ANC [42].

We used the modified Kessner Index with the aim of better evaluating ANC [12, 23, 33]. The adequacy of number of visits by gestational age was used incorporated into the index in this study, since preterm gestation should not be compared with term gestation for the number of visits. Even so, no association was found between the modified Kessner Index and LBW.

In other studies conducted in Brazil in which the same indices were used, some authors reported ANC adequacy of 10.7% for the LBW group [4], while others reported values of 38.4% and 60% for this parameter [43, 44]. The last two studies used the same index as our study, although with no specific differentiation for their relationship with the birth weight.

In this study, the number of visits by gestational age was associated with LBW. This association was found through the multivariable statistical analysis, and it was the contributing factor for the reduction in adequacy of ANC. The early initiation of ANC and adequate number of visits enables access to diagnostic and therapeutic methods for several pathologies that have serious repercussions on newborn and maternal health [45]. Our study showed that late initiation of ANC was neither a contributing factor for reducing ANC adequacy nor related to LBW.

We would like to point out that ANC protocols from other countries recommend that ANC begin prior to 12 weeks gestation [46], which could contribute to the ANC inadequacy observed in both of our study groups.

In Brazil, the interface between the public and private healthcare sectors has evolved over time, and yet it remains a constant source of conflict [47, 48]. The mixture of public and private investment in healthcare services also leads to distortions in the use of procedures according to how much the government will reimburse private providers for a specific intervention [49].

In our study, one of the explanations for the low ANC adequacy indices could be that many mothers begin ANC using a private health care plan, and then complete it in a BHU or CH, so that the childbirth is funded by the Public Health System. A likely reason for this is that some private health plans in Brazil cover only medical visits. This process could lead to underreporting of antenatal visits, as well as to the late initiation of ANC documented in this study. There is a need for better evaluation to confirm that this scenario occurs.

ANC consists of many items, and there is a chain of events related to a basic prerequisite, that is, the early initiation of follow-up by the pregnant woman, which would enable monitoring of the proposed actions. The proposed actions will lead to high quality ANC, and as a result, pregnancies with good outcomes. Therefore, the great challenge to be overcome by ANC providers is to make available the set of activities proposed for all women in a timely fashion, so that good outcomes are reached with a reduction in the rate of LBW.

Higher adequacy of number of visits adjusted to gestational age and ANC laboratory studies and exams summary measure in prenatal care performed in the primary health care units shows that a close bond between the pregnant women and the BHUs has led to an early starting of follow up, and as a consequence, a higher number of visits and exams as suggested by the PPBH, whereas prenatal care of high-risk pregnant women were referred to the secondary level of health care. Therefore, the limitations of this study could be the lack of differentiation between these measures according to period of gestation at the beginning of prenatal follow-up, and a more individualized analysis of exams in the secondary level of health care. The number of visits were not considered in this analysis and exams previously performed in the primary level of health care. The closer bond between the pregnant women and the BHU may have kept some pregnant women in the ANC follow up at this level of health care, even being referred to the secondary level of health care. The significantly higher adequacy of both measures for the Control group in primary health care level, and significantly higher adequacy for the Case group in secondary health care level are not unexpected findings - since pregnancies in the case group have already been identified as being at high risk, and should be receiving a different care pathway in more specialized level of care.

A number of questions arise from the findings presented: Because primary health care is equipped with well-defined technology for ANC, which is clearly established in the SUS in Brazil, why do we still find low ANC adequacy indices? Do health services have a sufficient number of skilled professionals or is a lack of sufficient human resources contributing to low ANC adequacy? Despite the established protocols, are there barriers to ANC access in primary care?

The PPBH has been used in Brazil since 2000, and an effective monitoring of its actions has been held through the SISPRENATAL. Thus, it is considered that a good level of awareness of its practices exists among health professionals who work in primary health care in the SUS [50]. Another question which arises from the findings of this study concerns the reason why health professionals of ANC do not follow the guidelines. Is there a lack of knowledge regarding their importance? Should there be better professional training in this guideline?

Striking improvement in access to services and in coverage levels in Brazil has been reported for most health interventions, but it has also been pointed out that the quality of services provided by the SUS is sometimes below the expected level, for example, concerning ANC [49]. Poor quality of care is related to institutional issues, such as high turnover of health workers in primary health care, and difficulties in recruiting skilled physicians for small cities that are removed from major urban centers [51]. Likewise, Brazilian studies conducted in some mid-sized and large cities have reported too many medical interventions in obstetric care for vaginal births. At the same time, the use of labor monitoring, such as partograms, and measurement of maternal arterial blood pressure and fetal heartbeats is low [52, 53]. The failure to measure maternal blood pressure and auscultate fetal heartbeats reveals a major technical failure in the care process, contrary to the purpose of decreasing maternal and neonatal mortality. Changes in these parameters show the need to hasten the delivery or to transfer the patient to a more complex level of care. Also, heartbeat auscultation is essential for evaluation of fetal vitality and subsequent conduct [52].

Cases of difficult access and fragmentation between primary (antenatal) care and hospital (birth) care have been reported [53]. Even in cities like Botucatu, a reference center because of the existing Public University Hospital – CH, the turnover rate of health professionals is high in the BHUs, which may jeopardize access to or quality of ANC.

Cesarean section rates in Brazil have increased steadily, and today they account for more than half the deliveries, although WHO recommendations based on medical criteria are of 15% [54]. This finding may be explained by non-medically indicated cesareans, which show non clinical factors having greater importance than clinical factors in cesarean indices in Brazil. One of the consequences of non-medically indicated cesareans is the increase in prematurity [30]. In 2009, for the first time, the number of newborns born by this type of procedure was greater than those born by vaginal births. Cesarean section is associated with several adverse effects on women and newborns [27]. Recent evidence suggests that increased rates of preterm births and LBW in Brazil are associated with increased rates of cesarean sections and labor induction, and they may be associated with an increase in severe near-miss neonatal morbidity [27]. This discussion was previously inserted in the study on risk factors for LBW, as the authors reported higher occurrence of cesarean sections in the LBW group than in the adequate weight group. Cesarean sections were more often performed in the CH, in which deliveries with a higher risk of maternal or fetal complications at birth occurred [55]. The place of delivery could be justified as a search for better perinatal outcomes because university hospitals are able to provide the intensive neonatal care usually required by these newborns [56].

According to the National Commission on Social Determinants of Health, the effects of the educational level are expressed in different ways, such as in the perception of health problems; ability to understand health information; adoption of healthy life styles; use of health services, and compliance with therapeutic procedures. The performance of preventive exams for breast and cervix cancer as well as prenatal visits are good examples of inequalities related to access and use of health services according to the level of education [57]. Maternal education, available in the data source of this study, was the only socioeconomic indicator evaluated, and no statistically significant difference was found concerning this parameter between the groups. Thus, according to the socioeconomic status, there was no interference in the access to exams considering the two groups . An important point in the access to better resources in this study are the fundamentals of the public health system in Brazil, the SUS, which makes the access to health care services and exams available in its entirety and for all, at different levels of health care regardless of socioeconomic status.

We consider that for a more comprehensive discussion on social causes of inequalities of Health in Brazil and their relationship with LBW, a better characterization of the socioeconomic status of the pregnant women population would make contributions to further studies.

The authors of this study decided to make up the third quality criterion of ANC including the ultrasound exam. Despite controversies of using it as a routine exam, as there is no evidence-based recommendation, this exam was included as a routine procedure in the Basic Units in the study city according to the Municipal Protocol [14]. Antenatal care of high-risk pregnant women was performed in the CH, for whom the ultrasound is considered a routine and complementary exam [13].

Our study has a number of strengths and some limitations, such as the lack of medical records on prenatal follow-ups, initiation of ANC, laboratory studies and exams. We believe these limitations had no effect on the results, as they were equally distributed between both groups. Also, despite the fact that the SUS in Brazil provides free access and is available to all the population, a segment of the Brazilian population receives ANC in a private health care setting. The study of ANC in this environment can contribute further insight into the relationship between ANC adequacy and LBW.

The adjusted multivariable analysis of LBW risk factors including maternal smoking, among others, was conducted and discussed by the authors elsewhere, and no association between inadequate ANC and LBW was found [55].

We believe that other institutions in middle- and low-income countries could use the methodology applied in this study to gain insight into the relationship between ANC and LBW. Such uptake would be a valuable contribution to research into maternal-infant health, and assist in the promotion of equity in health.

## Conclusions

Our data support the association between an inadequate number of prenatal visits and ANC laboratory studies and exams with increased risk of LBW newborns. It also includes low indices of coverage for basic actions already well regulated in the SUS in Brazil.

Despite the association found in the study, we cannot conclude that LBW would be prevented only by an adequate ANC, as LBW is associated with factors of complex and multifactorial etiology.

The results could be used to develop monitoring measures and evaluation programs of health care assistance during pregnancy, at delivery and to newborns, focusing on a reduction in LBW rates.

In Brazil, there is a need for integrated strategies to promote social demographic and cultural development of women’s reproductive health, but not only during pregnancy. Accordingly, higher levels of awareness of ANC and its improvement of maternal and infant health could be reached. Further studies on the evaluation of quality of ANC experienced by women associated with better evaluation of barriers to ANC access could bring some improvement on this issue.

*Acronym is based on the Portuguese-language name.

## Abbreviations

ANC: Antenatal care
LBW: Low birth weight
PPBH: Program for Prenatal and Birth Care Humanization
SGA: Small for gestational age
SUS*: Unified Health System
SINASC*: Information system of live births
LBC: Live birth certificate
BHU: Basic Health Unit
CH: Clinics Hospital
SEADE*: State System Foundation for Data Analysis
DATASUS: Informatic Department of SUS*
SAS: Statistical Analysis System
REC: Research Ethics Committee
SE: Socioeconomic Status
US: Ultrasound exam.
